# Supplementary material for: From sequence to enzyme mechanism using multi-label machine learning
Source: BMC Bioinformatics. 2014 May 19;15:150. doi: 10.1186/1471-2105-15-150 (PMC4229970; doi:10.1186/1471-2105-15-150)
Supplement: Additional file 2 — Java code of ml2db. Additional file ml2db_code.tar.gz contains the Java source code to run the multi-label machine learning experiments and save the results to database. The code’s Javadoc is included. [file 1471-2105-15-150-S2.zip › additional file 2/ml2db/ml2db/doc/test/ml2db/al/utility/globaldistance/DistancesCalculatorTest.html]

DistancesCalculatorTest


JavaScript is disabled on your browser.


- Overview
- Package
- Class
- Use
- Tree
- Deprecated
- Index
- Help

- Prev Class
- Next Class

- Frames
- No Frames

- All Classes

- Summary:
- Nested |
- Field |
- Constr |
- Method

- Detail:
- Field |
- Constr |
- Method


test.ml2db.al.utility.globaldistance

## Class DistancesCalculatorTest

- java.lang.Object
- - junit.framework.Assert
  - - junit.framework.TestCase
    - - test.ml2db.al.utility.globaldistance.DistancesCalculatorTest

- All Implemented Interfaces:
  :   junit.framework.Test

  ---

    

  ```
  public class DistancesCalculatorTest
  extends junit.framework.TestCase
  ```

  Class

  Version:
  :   10 May 2012

  Author:
  :   Luna De Ferrari ldf(at)st-andrews.ac.uk or
      luna.deferrari@st-andrews.ac.uk

- - ### Constructor Summary

    Constructors

    Constructor and Description

    `DistancesCalculatorTest()`
  - ### Method Summary

    Methods

    | Modifier and Type | Method and Description |
    | --- | --- |
    | `static DistancesCalculator` | `getDistancesCalculatorOnTestSet()` |
    | `void` | `testCalculateAllDistances()` Test method for `DistancesCalculator.calculateAllDistances()` . |
    | `void` | `testSerializeObject()` Test method for `main.ml2db.Serializer#serialize(java.lang.Object, java.lang.String)` . |

    - ### Methods inherited from class junit.framework.TestCase

      `countTestCases, getName, run, run, runBare, setName, toString`
    - ### Methods inherited from class junit.framework.Assert

      `assertEquals, assertEquals, assertEquals, assertEquals, assertEquals, assertEquals, assertEquals, assertEquals, assertEquals, assertEquals, assertEquals, assertEquals, assertEquals, assertEquals, assertEquals, assertEquals, assertEquals, assertEquals, assertEquals, assertEquals, assertFalse, assertFalse, assertNotNull, assertNotNull, assertNotSame, assertNotSame, assertNull, assertNull, assertSame, assertSame, assertTrue, assertTrue, fail, fail, failNotEquals, failNotSame, failSame, format`
    - ### Methods inherited from class java.lang.Object

      `equals, getClass, hashCode, notify, notifyAll, wait, wait, wait`

- - ### Constructor Detail


    - #### DistancesCalculatorTest

      ```
      public DistancesCalculatorTest()
      ```
  - ### Method Detail


    - #### testCalculateAllDistances

      ```
      public void testCalculateAllDistances()
      ```

      Test method for
      `DistancesCalculator.calculateAllDistances()`
      .


    - #### testSerializeObject

      ```
      public void testSerializeObject()
      ```

      Test method for
      `main.ml2db.Serializer#serialize(java.lang.Object, java.lang.String)`
      .


    - #### getDistancesCalculatorOnTestSet

      ```
      public static DistancesCalculator getDistancesCalculatorOnTestSet()
      ```


- Overview
- Package
- Class
- Use
- Tree
- Deprecated
- Index
- Help

- Prev Class
- Next Class

- Frames
- No Frames

- All Classes

- Summary:
- Nested |
- Field |
- Constr |
- Method

- Detail:
- Field |
- Constr |
- Method
